# Problem-Solving and Tool Use in Office Work: The Potential of Electronic Performance Support Systems to Promote Employee Performance and Learning

**DOI:** 10.3389/fpsyg.2022.869428

**Published:** 2022-04-29

**Authors:** Tamara Vanessa Leiß, Andreas Rausch, Jürgen Seifried

**Affiliations:** Mannheim Business School (MBS), University of Mannheim, Mannheim, Germany

**Keywords:** electronic performance support systems (EPSS), learning at work, informal learning, knowledge sharing, problem-solving, tool use, enterprise resource planning (ERP)

## Abstract

In the context of office work, learning to handle an Enterprise Resource Planning (ERP) system is important as implementation costs for such systems and associated expectations are high. However, these expectations are often not met because the users are not trained adequately. Electronic Performance Support Systems (EPSS) are designed to support employees’ ERP-related problem-solving and informal learning. EPSS are supposed to enhance employees’ performance and informal workplace learning through task-specific and granular help in task performance and problem-solving. However, there is little empirical research on EPSS. Two survey studies addressed this research gap. In the first study, 301 people working in Human Resource (HR)-related positions and functions evaluated the learning potential of EPSS as well as potential advantages and obstacles concerning the implementation and use of EPSS. Though other measures are currently assessed as more important for learning, HR employees expect a strong increase in the significance of EPSS for employee learning. In the second study, 652 users of ERP software completed a questionnaire on characteristics of their daily work tasks, team characteristics, individual dispositions, their coping with ERP-related problems, and characteristics of EPSS. Findings indicate that the most frequently available and used approach when dealing with an ERP-related problem is consulting colleagues. Three EPSS types can be distinguished by their increasing integration into the user interface and their context-sensitivity (external, extrinsic, and intrinsic EPSS). While external and extrinsic EPSS are available to many users, intrinsic EPSS are less common but are used intensively if available. EPSS availability is identified to be a strong positive predictor of frequency of EPSS use, while agreeableness as well as the task complexity and information-processing requirements show small negative effects. Moreover, more intensive ERP users use EPSS more frequently. In general, ERP users value, features such as context-sensitivity, an integration of the EPSS into the ERP system’s user interface, the option to save one’s own notes, and information displayed in an extra window. It is expected that EPSS will play an important role in workplace learning in the future, along with other measures.

## Introduction

In this paper, we investigate the significance of Electronic Performance Support Systems (EPSS) for informal workplace learning, including their actual availability and frequency of use among different ERP user types. Office workplaces are shaped by two main developments. Firstly, knowledge workers in office workplaces are confronted with increasingly complex tasks because routine activities are automated or outsourced. Hence, more complex tasks remain for which routine solutions are not available (Littlejohn and Margaryan, 2014; Frey and Osborne, 2017; Bughin et al., 2018). Secondly, more and more software is used at office workplaces for organizational operations and decision-making (Venkatesh and Bala, 2008; Eikhof, 2012; Billett, 2021). Therefore, the skills needed in working life are increasingly linked to “electronically mediated tasks and work roles” (Billett, 2021, p. 1). Thus, an essential part of knowledge workers’ competence is mastering the handling of software tools (Warren et al., 2009; Hämäläinen et al., 2018). Säljö (1999) argues that any learning means learning to use tools. His concept of cultural tools comprises not only physical tools but also intellectual concepts, such as technical language or specific calculation schemes and, of course, software tools. Similarly, Engeström (1993), based on Vygotsky’s (1978) cultural-historical activity theory, emphasizes the significance of tools as mediating artifacts between the subject (i.e., the employee) and the object (i.e., the task at hand) and outlines that these tools can be physical or symbolic, internal or external. In case of office work, software applications are the most important tools. One important category of software applications in office work are Enterprise Resource Planning (ERP) systems. ERP systems usually comprise a variety of software modules that integrate data from several departments into one single system and support the management of all business processes (Kalling, 2003; Nwankpa, 2015). Learning in the context of an ERP system is especially of interest because of two reasons. First, as costs of implementing an ERP system are high, so are the expectations of the increase in the performance. However, these expectations are often not met because the users are not capable of handling these systems and not trained adequately (Jasperson et al., 2005; Rezvani et al., 2017). Second, the transfer of formally acquired knowledge to one’s workplace often proves difficult for employees (Chang, 2004; Mao and Brown, 2005; Nguyen and Klein, 2008; Nguyen, 2009). This is also true for formal learning regarding ERP and sheds light on the importance of post-implementation learning, which means continuous on-the-job learning after an information technology has been implemented (Deng, 2000; Chou et al., 2014). In this context, informal learning plays an important role, as most learning in the workplace occurs informally (Eraut, 2010). Informal learning in general can be defined as “any kind of learning which does not take place within, or follow from, a formally organised learning programme or event” (Eraut, 2000, p. 114). According to Eraut (2000, 2004), informal learning can include different modes of learning, from unconscious learning (i.e., *implicit learning*) to conscious non-formal learning with clear learning objectives and time set aside to pursue it (i.e., *deliberative learning*). A typical working activity where learning is seen as a possible and welcome by-product is problem-solving (Eraut, 2000, 2004).

To support these different modes of informal workplace learning, contextual performance support, community or social technologies and adaptive learning technologies seem promising (Lindstaedt et al., 2010; Ley et al., 2014; Li and Herd, 2017; Kravčík, 2019; Ley, 2020). A solution that integrates these approaches and provides instant performance, and learning assistance when using software tools (e.g., ERP systems) and solving problems are EPSS (Chang, 2004). EPSS has the potential to “provide the right information to the right user at the right time” (Nguyen, 2009, p. 95). The concept of EPSS has its roots in the 1990s. Gery (1991) first mentioned EPSS and later identified 19 attributes of performance-centered EPSS (Gery, 1995). These included for example “establish and maintain a work context” or “contain embedded knowledge in the interface, support resources, and system logic” (Gery, 1995, p. 53). A more contemporary definition describes EPSS as “an electronic infrastructure that captures, stores, and distributes individual and corporate knowledge assets throughout an organization to enable individuals to achieve required levels of performance in the fastest possible time and with a minimum of support from other people” (Noe, 2017, p. 368). In a nutshell, granular task-specific information is presented to solve a problem at hand (Mao and Brown, 2005). Hence, performance is supported during work (Gery, 1995; Nguyen and Klein, 2008) at all career stages, ranging from “day-one performance” in rookies (Gery, 1995, p. 48) to the attainment of expert performance (Clem, 2007). EPSS reduce cognitive load (Tamez, 2012) and serve as an extension of the employees’ long-term memory (Bastiaens et al., 1997; Mao, 2004). This means that the necessary knowledge may have been learned by an employee before but has not been memorized or has been forgotten in the meantime. However, several authors stress the potential of EPSS to not only enhance performance and remind users of what they have learned beforehand but also to support informal learning in the workplace (Gery, 1995; Raybould, 1995; van Schaik et al., 2002), for example by providing scaffolding (Cagiltay, 2006) or synthesizing and reflecting (Hung and Chao, 2007).

**Figure 1:**
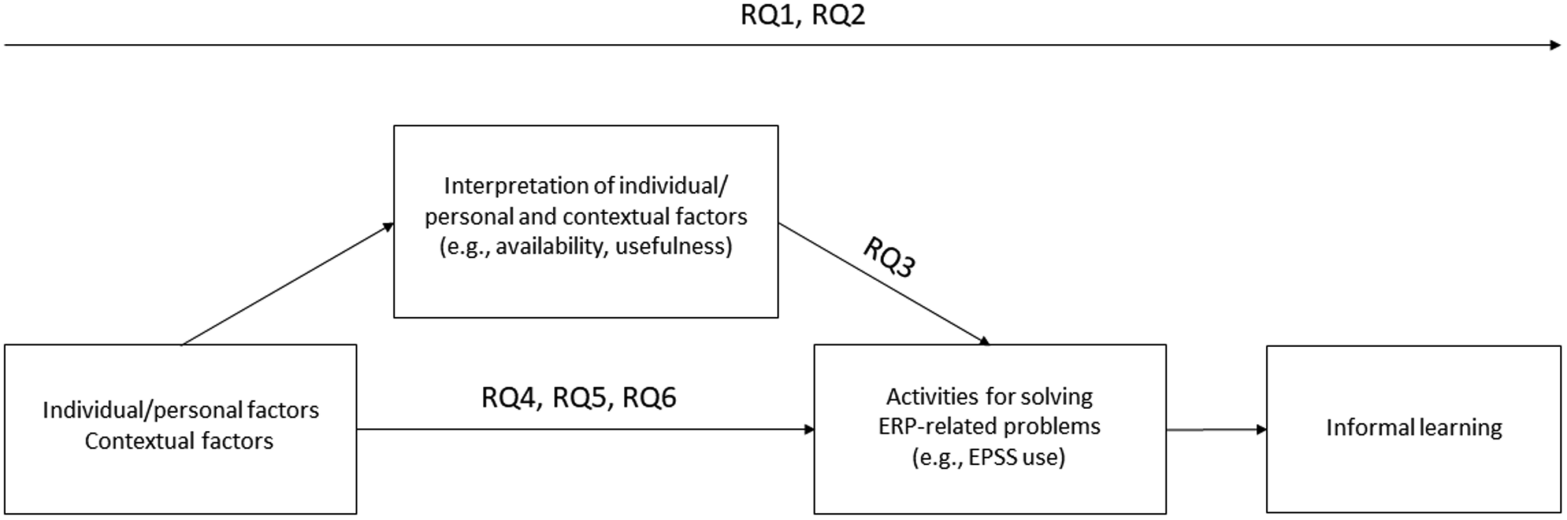
Conceptual model of the investigated research questions.

Although companies have been applying EPSS—with varying success—since the 1990s, empirical research on their effectiveness is scarce (Chang, 2004; Mao, 2004; Mao and Brown, 2005; Nguyen and Klein, 2008; Gal and Nachmias, 2012; Gal et al., 2017). This is especially true for recent studies that have included new technological capabilities in their definition and design of EPSS. In addition, some of the results of older studies can now be considered obsolete, because technologies available in the past are very different from those available today (Ley, 2020). Moreover, literature on EPSS is criticized for not being empirical (Mao, 2004; Nguyen et al., 2005; Gal and van Schaik, 2010) but based instead on anecdotal evidence (Mao, 2004; Gal and van Schaik, 2010). The present exploratory studies address this research gap from two perspectives. First, the potential of EPSS is assessed more generally by people working in Human Resource (HR)-related positions and functions (= HR employees) (RQ1 and RQ2). Second, the user perspective is taken into account (RQ3 to RQ6). In addition, EPSS can be viewed from two perspectives. First, EPSS can be viewed as a resource created to support employees’ performance, problem-solving and learning. This is a more general view of EPSS, which can also address their availability as well as the design and different characteristics of a supplied EPSS. Second, the actual use of EPSS and its results can be examined. We considered these two perspectives in our studies. Altogether, we investigated six research questions, which are also illustrated in Figure 1.

- RQ1: How significant are EPSS considered as a learning resource at present and in future by HR employees?
- RQ2: What potential advantages and obstacles concerning the implementation and use of EPSS are seen by HR employees?
- RQ3: What activities are available to ERP users when they need to solve an ERP-related problem in the workplace and how frequently are these activities used when available?
- RQ4: Do the ERP user types differ in terms of availability and frequency of EPSS use when dealing with an ERP-related problem in the workplace?
- RQ5: What factors (contextual and individual/personal factors) influence the frequency of EPSS use when dealing with an ERP-related problem in the workplace?
- RQ6: Which EPSS characteristics are considered the most useful by ERP users and do ERP user types differ in their assessment of usefulness?

In order to systematize the hypothetical influencing factors, a comprehensive Model of Informal Workplace Learning Through Problem-Solving was developed in a first step. Based on that, two studies were conducted in order to answer the research questions. In study 1, 301 HR employees completed a questionnaire on the significance of EPSS for corporate learning as well as potential advantages and obstacles. In study 2, 652 users of ERP systems completed a questionnaire on their use of ERP systems, the availability, and their use of activities for solving ERP-related problems, their evaluation of EPSS characteristics as well as contextual and individual factors.

## Electronic Performance Support Systems and Informal Workplace Learning

### Types, Effects, and Applications of EPSS

In general, three types of EPSS can be distinguished, which differ primarily in the degree of their integration into the target system (e.g., ERP systems) and their context-sensitivity (Gery, 1995). (1) External performance support is not integrated into the system or the work interface and can also be paper-based, for instance. As such, users have to turn away from the target system and to break the work context in order to use the external EPSS (Gery, 1995; Mao, 2004; Sumuer and Yildirim, 2015). Early examples of external performance support are help desks, questions and answers Q&A, job aids, manuals, knowledge databases, and search engines (Gery, 1995; Nguyen et al., 2005; Nguyen and Hanzel, 2007; Gal and Nachmias, 2012). More recent examples also include Web 2.0 technologies, such as online forums and communities and the content provided there. (2) Extrinsic performance support is integrated into the system, but not into its primary user interface (Gery, 1995). Instead, the presumably helpful information is displayed outside of the target system (Nguyen et al., 2005). This means that, for instance, a new window is opened. The system is often context-sensitive, which means that it can identify which task the user is working on. Based on this information, the extrinsic system can suggest appropriate information (Nguyen et al., 2005). Examples for extrinsic EPSS are advisors, wizards, and cue cards (Gery, 1995), but also often the conventional help function within a software. (3) Intrinsic EPSS integrate granular and context-sensitive information into the target system’s user interface (Gery, 1995). Hence, the information is provided directly in the flow of work (Nguyen et al., 2005; Gal and van Schaik, 2010; Gal and Nachmias, 2012). For users, it is often difficult to distinguish between the target system itself and the EPSS (Gery, 1995). An example more in line with older notions of an intrinsic EPSS is the integrated help that is displayed automatically when creating a new title within the reference management software Citavi^®^ (Swiss Academic Software, Switzerland). More recent technical features that can be assigned to either extrinsic or intrinsic EPSS, depending on their design, include videos that colleagues have recorded about their own activities in the system as well as tutorials or guided tours, for example by the ERP vendor. In addition, the possibility to take notes in the system that are displayed to the documenting person or to groups of people, the next time this step in the system is entered, is conceivable. Other possible options may include social technologies, such as an integrated chat function for direct questions to experts or suggested experts with contact details. While Gery (1995) initially meant this distinction as a hierarchy with intrinsic EPSS as the superior type, in our opinion, today’s technological developments question this general superiority. Newer EPSS and EPSS characteristics, such as video platforms for tutorials, can also be very effective, although they fall into the categories of external or extrinsic EPSS. The effectiveness depends more on the specific design of the EPSS and its characteristics than, for example, on the way they are integrated into the user interface alone. Therefore, we still find Gery’s (1995) types useful to classify EPSS and EPSS characteristics, but we no longer assume a hierarchy in quality.

Overall, in our opinion, a contemporary definition of EPSS should be a much broader and more flexible one, that includes all technological devices and applications that enable users to solve problems in real time and thus enable learning in the flow of work. This is consistent with Hannafin et al.’s (2002, p. 100) conclusion that EPSS do not have fixed features or components but can be seen more as “a perspective on designing systems that support learning and/or performing”. Against this background, EPSS are still very relevant to address highly recent problems. They already contained the first approaches to adaptivity and context-sensitivity, that are still considered central in many current approaches, at an early stage. Today, thanks to new technological possibilities, they can be extended by numerous functionalities and realize the early goals much more effectively and successfully than in early implementations.

One of the most frequently mentioned benefits of EPSS is its potential to support employee performance (Barker and Banerji, 1995; Gery, 1995; Chang, 2004; Nguyen and Klein, 2008) and as a result different aspects of employee productivity (Bastiaens, 1999; Altalib, 2002). Several empirical studies have reported positive effects of EPSS on various measures of performance (Bastiaens, 1999; van Schaik et al., 2002; Mao and Brown, 2005; Nguyen et al., 2005; Gal and Nachmias, 2011; Lanese and Nguyen, 2012; Rios et al., 2013; Nuss et al., 2014; Yakin and Yildirim, 2016; Gal et al., 2017; Ugur-Erdogmus and Cagiltay, 2019). These were, for instance, positive effects on expertise reports or speed of task completion of police officers in Turkey (Yakin and Yildirim, 2016) and positive effects on time used for and quality of maintenance procedures of the engine air bleed system on a Boeing 737 aircraft (Rios et al., 2013). Some studies compared the effect of EPSS with traditional training and found EPSS to be at least partly superior (Bastiaens et al., 1995; Mao and Brown, 2005; Gal et al., 2017). Moreover, a few studies have investigated the effects of different EPSS types (external, extrinsic, and intrinsic EPSS) on employee performance and productivity (Nguyen, 2005; Nguyen et al., 2005; Gal and Nachmias, 2011; Yakin and Yildirim, 2016). These were, for instance, employees’ time on task and the service quality in a service call (Gal and Nachmias, 2011) and the performance in a task scenario within a company’s learning management system (Nguyen et al., 2005). The results of these few studies are ambiguous and no general superiority of one EPSS type over other types can be inferred. As already mentioned, however, we believe that in studies that used more recent technological possibilities, such a general superiority of one type is not to be expected.

### The Role of EPSS in Informal Workplace Learning

In addition to enhancing performance, EPSS are also supposed to foster (informal) workplace learning (Gery, 1995; Raybould, 1995; van Schaik et al., 2002; Mao, 2004; van Schaik, 2010; Kert and Kurt, 2012; Kalota and Hung, 2013; Gal et al., 2017). This is possible through different aspects and functionalities of EPSS. EPSS deliver just enough granular knowledge for the task at hand. Hence, compared to comprehensive formal training, the problems of inert knowledge and inhibited learning transfer are reduced since the newly acquired knowledge is immediately applied (Mao and Brown, 2005). In this context, EPSS can either replenish formal training or even substitute formal training in some cases (Mao, 2004; Mao and Brown, 2005; Nguyen and Klein, 2008; Noe, 2017). In particular, EPSS can support occasional users that would not benefit from extensive training in advance because most of the acquired knowledge would have faded before its application (Mao and Brown, 2005). Furthermore, EPSS can reduce cognitive load (Tamez, 2012) and provide scaffolding during complex tasks (Mao and Brown, 2005). Indeed, the few empirical studies on EPSS and workplace learning report positive effects (Wild, 2000; van Schaik et al., 2002; Mao and Brown, 2005; Gal and Nachmias, 2011; Kert and Kurt, 2012; Kalota and Hung, 2013; Nuss et al., 2014). Another research project in the context of computer-mediated work included some adaptive and performance support functionalities, however, the authors did not call them an EPSS. Within the project, APOSDLE context-sensitive help and information as well as relevant experts regarding the working tasks at hand were suggested (Lindstaedt et al., 2010). The authors also reported a positive effect on the knowledge of knowledge workers in highly specialized domains, however not in broad customer-driven domains.

EPSS primarily support informal learning through solving task-related problems during the flow of work (Barker and Banerji, 1995; Mao, 2004). Since problems are defined as a situation in which an individual lacks the knowledge to achieve a current goal (Newell and Simon, 1972), problem-solving requires searching for information and hence, enables the acquisition of new knowledge. According to Rausch’s (2011) and Rausch et al.’s (2015) classification of Approaches to Problem-Solving in the Workplace, solution approaches are based on either mental models or real-world experiences, and they are developed on either one’s own or adopted from someone else (see Table 1; similar activities are reported by Cuyvers et al., 2016). This matrix is meant to be conceptually exhaustive but, of course, further examples could be listed. However, in most problem situations, people will not only use one approach but instead utilize combinations of different approaches that will usually start with reflection on the problematic situation.

**Table 1:** Approaches to problem solving in the workplace (Rausch, 2011, p. 98; Rausch et al., 2015, p. 452).

	Approaches based on mental models	Approaches based on real-world experience
Development of one’s own approach	Reflecting (e.g., mental simulation, interpolation, analogy, abstraction, reduction)	Trying out (e.g., experimentation, hypothesis testing, trial and error learning)
Adoption of someone else’s approach	Consulting competent others (e.g., assistance, guidance, instruction, EPSS) Consulting codified information (e.g., guidelines, manuals, EPSS)	Observing competent others (e.g., observing role models, watching video tutorials, EPSS)

This classification of approaches again addresses the two perspectives in which EPSS can be viewed. On the one hand, EPSS’ use for problem-solving and informal learning can be considered. In the case of a software-related problem, for instance a problem regarding an ERP system, different examples for the approaches can be mentioned. Typically, problem-solving processes will start with a reflection on what is already known from prior experience and formal training. If combining this prior knowledge does not lead to a solution, one has to search for further information by using other approaches, for example by asking colleagues or reading the manual. In their diary study on everyday problem-solving in the domain of controlling, Rausch et al. (2015) found that asking colleagues was the most frequently applied strategy for novices but also for skilled employees. Consulting codified information, such as manuals, was used by novices but hardly used by skilled employees. It is a commonplace that people do not like to read manuals (Novick and Ward, 2006). On the other hand, EPSS can be seen as a resource that is designed and supplied to support employees. Thus, EPSS can be assigned to different approaches to problem-solving, depending on their design. For example, EPSS can enable employees to ask other people through a chat function integrated into the ERP system. EPSS can also provide codified information. For example, granular information that exactly matches the current task can be provided directly within the user interface. However, EPSS can also include multimedia content like short tutorials, again granular and matching to the problem at hand, or quick contact information about experts that can be approached. Moreover, they can provide videos of the current task that have been recorded by colleagues. In this way, others can be “observed” while performing the task. Thus, EPSS can support problem-solving processes and enable learning in a variety of ways.

### Model of Informal Workplace Learning Through Problem-Solving

**Figure 2:**
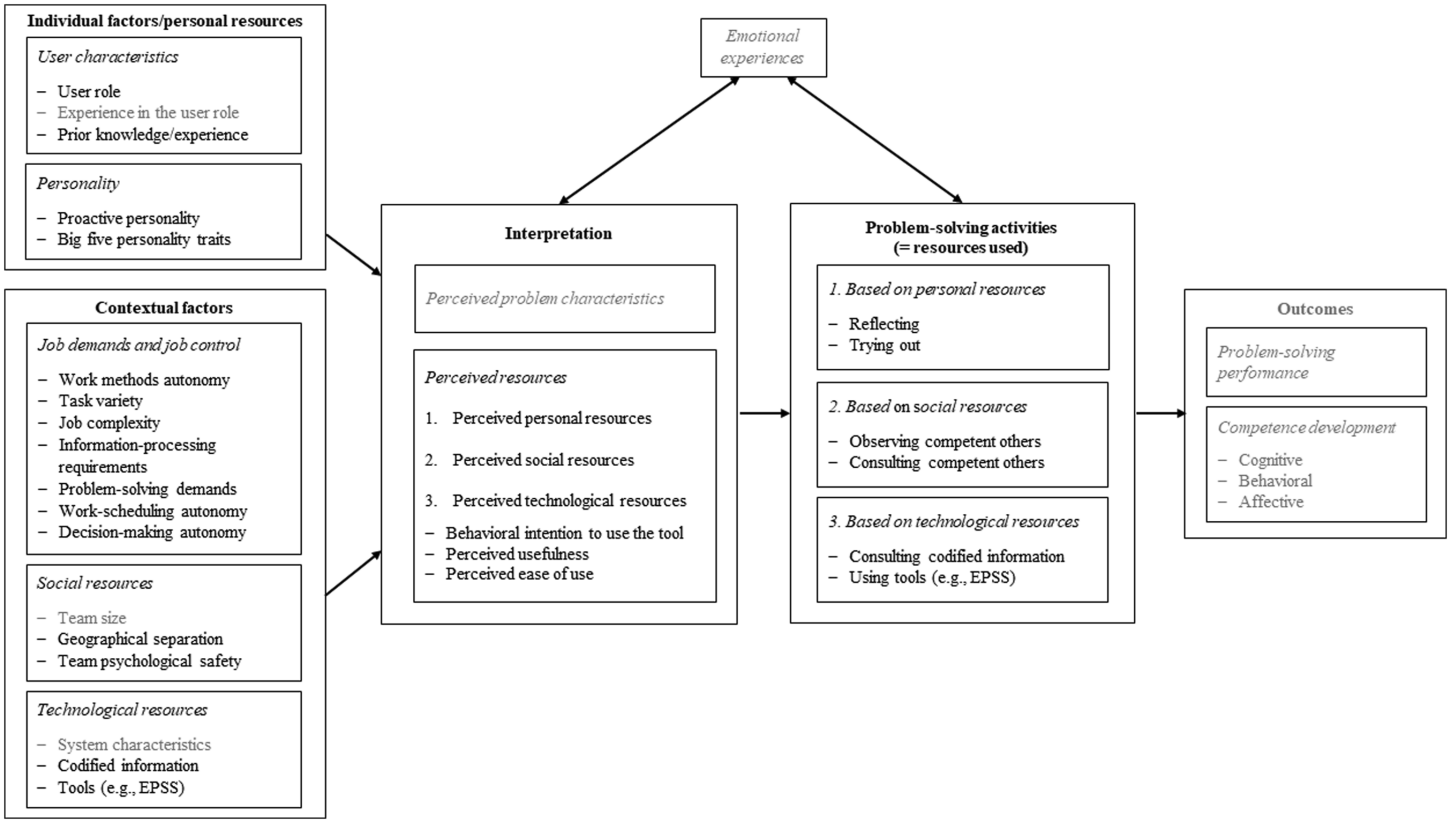
Model of informal workplace learning through problem-solving.

In order to investigate EPSS’ role in technology-related problem-solving, we developed a holistic model, as problem-solving is dependent on the person of the problem-solver and embedded in the organizational and social context. Figure 2 shows our model of Informal Workplace Learning Through Problem-Solving as a synthesis of several already existing other models. It combines basic assumptions of Tynjälä’s (2013) 3-P model, the Job Demand Control Support (JDCS) model (Karasek, 1979; Johnson and Hall, 1988; Karasek and Theorell, 1990), the Approaches to Problem-Solving in the Workplace (Rausch, 2011; Rausch et al., 2015), the Technology Acceptance Model (TAM 3; Davis et al., 1989; Venkatesh and Bala, 2008), and the Affective Events Theory (AET) by Weiss and Cropanzano (1996).

The basic structure of the model is based on Tynjälä’s (2013) 3-P model. Individual factors—which we also refer to as personal resources—and contextual factors influence through the process of interpretation, problem-solving activities, and the use of resources in this context. These problem-solving activities then may result in problem-solving performance as well as competence development. Both interpretation and problem-solving activities can be influenced by emotional experiences and can themselves influence emotional experiences. Relevant personal resources include user characteristics and personality traits. User characteristics can comprise for example the user role, experience in this role and prior knowledge or experience. There is empirical evidence that prior usage experience with a technology can influence technology use (Lee et al., 2003; Eckhardt et al., 2013) and that work experience can significantly negatively affect a technology’s perceived usefulness (Laumer et al., 2016). Prior knowledge is also an important antecedent of informal learning processes in general (Tynjälä, 2013; Cerasoli et al., 2018). Regarding personality, the big five personality traits were found to influence or moderate technology acceptance (Devaraj et al., 2008). The big five personality traits (Noe et al., 2013, 2014; Cerasoli et al., 2018) and a proactive personality (Carmeli et al., 2009; Noe et al., 2014) are important antecedents of informal workplace learning as well. A proactive person can be described as someone “who is relatively unconstrained by situational forces, and who effects environmental change” (Bateman and Crant, 1993, p. 105). Thus, we assume that this disposition may also influence the choice of problem-solving activities (e.g., asking colleagues). Empirical studies have shown that proactive personality is positively related to information exchange with other people (Gong et al., 2012).

Contextual factors include aspects of job demands and job control, aspects shaping social resources, and aspects shaping technological resources. There are several studies that found job characteristics, such as job demands and job control to be related to informal workplace learning (Rausch, 2013; Noe et al., 2014; Cerasoli et al., 2018). In our model, we included work methods autonomy, task variety, job complexity, information-processing requirements, problem-solving demands, work-scheduling autonomy, and decision-making autonomy. These are work task characteristics that are conducive to emotion and learning (Rausch, 2012). Autonomy is also an antecedent of a technology’s perceived usefulness and perceived ease of use (Arsal et al., 2009) as well as technology use (Ahuja and Thatcher, 2005). In our model, social resources include team size, a person’s potential geographical separation from his or her team as well as the team psychological safety. Empirical evidence on the influence of team size in the context of technology use is, for instance, provided by Bradner et al. (2005). Their results show that interactions between team members, the willingness to communicate with others in the team, and the use of communication technology in the team differ significantly between distributed teams of different team sizes. Furthermore, geographical and possibly associated temporal and perceived distance in virtual teams can influence for example the communication within teams as well as the synchronous availability of colleagues (Morrison-Smith and Ruiz, 2020). A study by Liu et al. (2021) showed that the geographical separation in online professional networks can lead to information cocoons within geographic regions. Based on this empirical evidence, we suppose in our model that geographic distance could have an influence on the preferred problem-solving activity. Moreover, team psychological safety, defined as “a shared belief that the team is safe for interpersonal risk taking” (Edmondson, 1999, p. 354), affects learning in the workplace (Edmondson and Lei, 2014; Frazier et al., 2017; Newman et al., 2017). We expect team psychological safety to also influence the choice of problem-solving activities, since, for example, a low team psychological safety, in a problem situation, could lead to the fact that asking colleagues and superiors is rather avoided. The model part of technological resources comprises system characteristics, codified information, and tools (e.g., EPSS). We expect the presence of these aspects of technological resources as well as their interpretation to influence their actual use, as it is suggested by TAM (Davis et al., 1989; Venkatesh and Bala, 2008). TAM’s assumed relationships were investigated many times empirically (Lee et al., 2003; Marangunić and Granić, 2015) and also in the context of learning technologies (Granić and Marangunić, 2019). This assumption already sheds light on another important aspect of our model. Contextual factors not only affect workplace learning directly (Cerasoli et al., 2018; Jeong et al., 2018; Rintala et al., 2019), but also indirectly through an individual’s interpretation (Tynjälä, 2013). In case of a problem within a current work activity, the given individual factors/personal resources and contextual factors are subjectively and maybe unconsciously interpreted in terms of potential personal, social, and technological resources. Based on cognitive and non-cognitive processes, one or more problem-solving activities can be applied. These problem-solving activities result from the given individual factors/personal resources and contextual factors and are conceptually based on the Approaches to Problem-Solving in the Workplace (Rausch, 2011; Rausch et al., 2015). In this vein, Carvalho (2019) found that the organizational environment, tool features, and task requirements were relevant factors for EPSS adoption and use. The use of one or more problem-solving activities ultimately results in outcomes, such as problem-solving performance and competence development (Tynjälä, 2013; Rintala et al., 2019), which can include cognitive as well as behavioral and affective aspects (Kraiger et al., 1993).

In the context of ERP-related problems, employees interpret their own user roles and competences, the characteristics of the present task, of their team, and their technological environment. One might, for instance, not trust his or her own competences and hence consult a colleague instead, while someone else might not consider his or her colleagues to be sufficiently competent or might not dare to bother them. Similarly, regarding technological resources, the availability, the perceived usefulness, and the perceived ease of use are important for the intent to utilize a software tool, such as an EPSS. Problem-solving is not a linear process. For instance, one might start reflecting on a problem confidently, but self-confidence decreases if no solution is in sight. This may lead to a re-interpretation of the technological resources or to overcoming the threshold to ask colleagues. Typically, more than one approach to problem-solving is applied. Once, a problem with the ERP system is resolved and given that the solution path is memorized, the same situation will not pose a problem in the future, hence, competence development has taken place.

Finally, we expect both, the interpretation and the problem-solving activities, to be influenced by emotional experiences. We base this assumption on empirical evidence on emotional experiences’ effect on workplace learning (Hökkä et al., 2020) as well as on technology acceptance constructs (Venkatesh, 2000; Lee et al., 2003) and technology use (Lee et al., 2003; Beaudry and Pinsonneault, 2010). In addition, we assume that an influence in the other direction is also plausible, since learning activities (Hökkä et al., 2020) and technology use (Loderer et al., 2020) can also have an impact on emotions.

We conducted two survey studies which are the first step in a larger research project. The first study addresses HR employees’ rating of EPSS as a learning opportunity. In this study, EPSS are viewed primarily as a technological resource designed to support employees. The second study focuses on ERP users’ experiences of EPSS in solving software-related problems and is based on the developed model. Here, EPSS are seen primarily in light of their actual use for solving ERP-related problems. The second study comprises different activities for solving ERP-related problems (e.g., EPSS use) that are based on the perception of the availability of the individual factors/personal resources and contextual factors. Therefore, not all aspects of the theoretical model are investigated empirically. Model components that are not part of the two questionnaire studies are grayed out in Figure 2.

## Materials and Methods

### Procedure and Sample

To address the research questions presented in the introduction, two questionnaire studies with different target groups were conducted. Thus, a cross-sectional research design was applied (Bickman and Rog, 2009). The first survey study addressed RQ1 and RQ2. A total of 301 HR employees participated, most of whom worked in Germany (*n* = 285). We drew a non-probability convenience sample, as we looked particularly for participants working in HR-related departments and functions (Henry, 2009). The majority of participants were recruited *via* mail and direct messages *via* LinkedIn. The participants worked in HR management (*n* = 104), HR development (*n* = 78), training and development (*n* = 77) and other areas.

The second survey study addressed RQ3 to RQ6. The questionnaire was completed by 652 ERP users, most of whom worked in Germany. Again, we drew a non-probability convenience sample, because we required participants with experience using an ERP system in different industries to take part in the study (Henry, 2009). The majority of participants were approached by a professional research institute. In addition, participants were recruited by open calls for participation *via* LinkedIn and other networks. In the sample, 284 persons were female and 365 persons were male. Participants were relatively evenly distributed across age intervals between 20–69 years and reported an average work experience of 17.5 years. A subsample of 28% of the participants reported that they were occasional ERP users who use the system, for example, to have their vacation approved, to submit a travel request, or for actions that only occur rarely. Half of the participants indicated that they were regular ERP end users who use the ERP system as part of their everyday work activities. Another 14% of the participants described themselves as experts, which means that they have the key user role and/or that they were the person in their team or department that is contacted for questions regarding the ERP system. The last user group comprised 9% who were administrators or SAP consultants. Administrators are responsible for the configuration and adaption of the ERP system. SAP consultants advise other companies regarding SAP software. We refer here to SAP because the company is the market leader for ERP systems and their systems are widely used in German-speaking countries. Table 2 provides an overview of all participants in both studies.

**Table 2:** Overview participants study 1 and study 2.

	Study participants	
	Participants study 1	Participants study 2
HR employees		
HR management	104	
HR development	78	
Training and development	77	
Other areas	26	
ERP users		
Occasional users		182
End users		320
Experts		91
Administrators or SAP consultants		59
**∑**	285	652

### Measures

All questionnaires were distributed in German and in English. However, most participants answered the German version. All translations were checked by an English native speaker. The items used in the two questionnaires are included in the Supplementary Materials.

#### Study 1: Questionnaire for HR Employees

##### Significance of Different Learning Measures for Employees

Participants rated the significance of six different measures (face-to-face training, coaching, e-learning, augmented reality/virtual reality (AR/VR), social software, EPSS) in their company at present and in the future (i.e., next 3–5 years) on a five-point Likert scale from 1 = *irrelevant* to 5 = *very relevant*.

##### Advantages and Obstacles Concerning the Implementation and Use of EPSS

Participants were requested to tick as many options as they wanted from a selection of eight potential advantages (e.g., “Reduction of search and problem-solving time”) and seven obstacles concerning the implementation and use of EPSS (e.g., “A digital help system will find little or no acceptance among employees”).

#### Study 2: Questionnaire for ERP Users

##### ERP User Type

At the beginning of the questionnaire, participants should assign themselves to the user types (1) occasional user, (2) end user, (3) expert, and (4) administrator or SAP consultant, each of which was described.

##### Self-Assessed Skills in Using the ERP System

The participants assessed cognitive, behavioral, and affective facets of using the ERP system (e.g., “When using the ERP system I feel very safe with the applications I need regularly” for the affective facet) on a five-point Likert scale from 1 = *not agree at all* to 5 = *strongly agree*. The scale comprised three items and its consistency was good (Cronbach’s alpha = 0.85).

##### Proactive Personality

Proactive personality was measured, using four of the five items, one slightly modified, from Goller (2017) (e.g., “I like to fight for my ideas, even against the resistance of others”), selected from the German version of the Proactive Personality Scale (Kaschube, 2003; Lang-von Wins and Triebel, 2005). The items were rated on a five-point Likert scale from 1 = *not agree at all* to 5 = *strongly agree*. The internal consistency was satisfactory (Cronbach’s alpha = 0.73).

##### Big Five Personality Traits

To reduce participant burden, each of the five personality traits was measured by only one item that included four adjectives (e.g., “extroverted, talkative, communicative, cheerful” for extraversion) based on Saucier’s (1994) Mini Markers and its German version by Weller and Matiaske (2009). The items were rated on a five-point Likert scale from 1 = *not agree at all* to 5 = *strongly agree*.

##### Characteristics of the Work Task

Task characteristics were measured, using selected items from Rausch (2012) that were answered on a five-point Likert scale from 1 = *not agree at all* to 5 = *strongly agree*. Four items were used to measure *task variety* (e.g., “At my workplace, I do a lot of different things”; Cronbach’s alpha = 0.79), four items for *job complexity* (e.g., “… my job requires that I only do one task or activity at a time”; Cronbach’s alpha = 0.80), four items for *information-processing requirements* (e.g., “… my job requires me to monitor a great deal of information”; Cronbach’s alpha = 0.79) and four items for *problem-solving demands* (e.g., “… my job involves solving problems that have no obvious correct answer”; Cronbach’s alpha = 0.75). *Autonomy* was assessed by four items. One item each covered work methods autonomy and work-scheduling autonomy and two items covered decision-making autonomy (e.g., “At my workplace I can plan how I do my work” for work-scheduling autonomy; Cronbach’s alpha = 0.79).

##### Geographical Separation

The participants indicated in one item whether they were usually geographically separated from the core of their team (e.g., other site or home office) and whether they were in home office recently due to the Corona pandemic (yes or no).

##### Team Psychological Safety

Team psychological safety was measured using the scale of Harvey et al. (2019) (e.g., “In my team people are usually comfortable talking about problems and disagreements”), that comprises four items. Again, the five-point Likert scale from 1 = *not agree at all* to 5 = *strongly agree* was used. The internal consistency was α = 0.74.

##### Availability of Problem-Solving Activities

The availability of problem-solving activities according to the above classification of Approaches to Problem-Solving in the Workplace (see Table 1) was measured by one single item on each activity (e.g., “At my workplace, if I have problems with the ERP system, I basically have the possibility to ask my colleagues for help”). With regard to our research focus, we included four items on potentially available EPSS features, that cover the three EPSS types external, extrinsic, and intrinsic. All items were answered on a five-point Likert scale from 1 = *not agree at all* to 5 = *strongly agree*.

##### Frequency of Use of Problem-Solving Activities

If a participant indicated that a problem-solving activity was at least partly available (from 3 = *partly* to 5 = *strongly agree*), then a further item “I often use this possibility” was administered and answered on a five-point Likert scale from 1 = *not agree at all* to 5 = *strongly agree*.

##### Perceived Usefulness of EPSS Characteristics

Regardless of their availability and frequency of use, participants were asked to rate the usefulness of various (hypothetical) characteristics of EPSS by six items. The self-developed items cover all three EPSS types (external, extrinsic and intrinsic) and are roughly based on Nguyen (2005). All items (e.g., “In the ERP system, you can use information provided next to the user interface of the ERP system to complete the current problem” for intrinsic EPSS) were rated on a five-point Likert scale from 1 = *not helpful at all* to 5 = *very helpful*.

### Statistical Analysis

To address the research questions, we applied various statistical methods. For RQ1, we calculated two one-way repeated measures analyses of variance (ANOVA) to determine if there were statistically significant differences between the learning measures’ current and future significance for employee learning. RQ2 was evaluated descriptively to identify which advantages and obstacles concerning EPSS were mentioned most frequently by the participants. For RQ3, we again calculated two one-way repeated measures ANOVAs to determine if there were statistically significant differences between the problem-solving activities’ availability and frequency of use. To investigate if the ERP user types differ in terms of availability and frequency of use of EPSS (RQ4), we calculated two one-way multivariate analyses of variance (MANOVA). RQ5 was investigated by a hierarchical multiple regression analysis to identify significant predictors of EPSS’ frequency of use. For RQ 6, a one-way repeated measures ANOVA was calculated to determine if there was a statistically significant difference between the perceived usefulness of the different EPSS characteristics. In addition, to investigate if the ERP user types differ in their assessment of the perceived usefulness, a one-way MANOVA was performed.

## Results

### Significance of EPSS as a Measure for Learning (RQ1)

**Figure 3:**
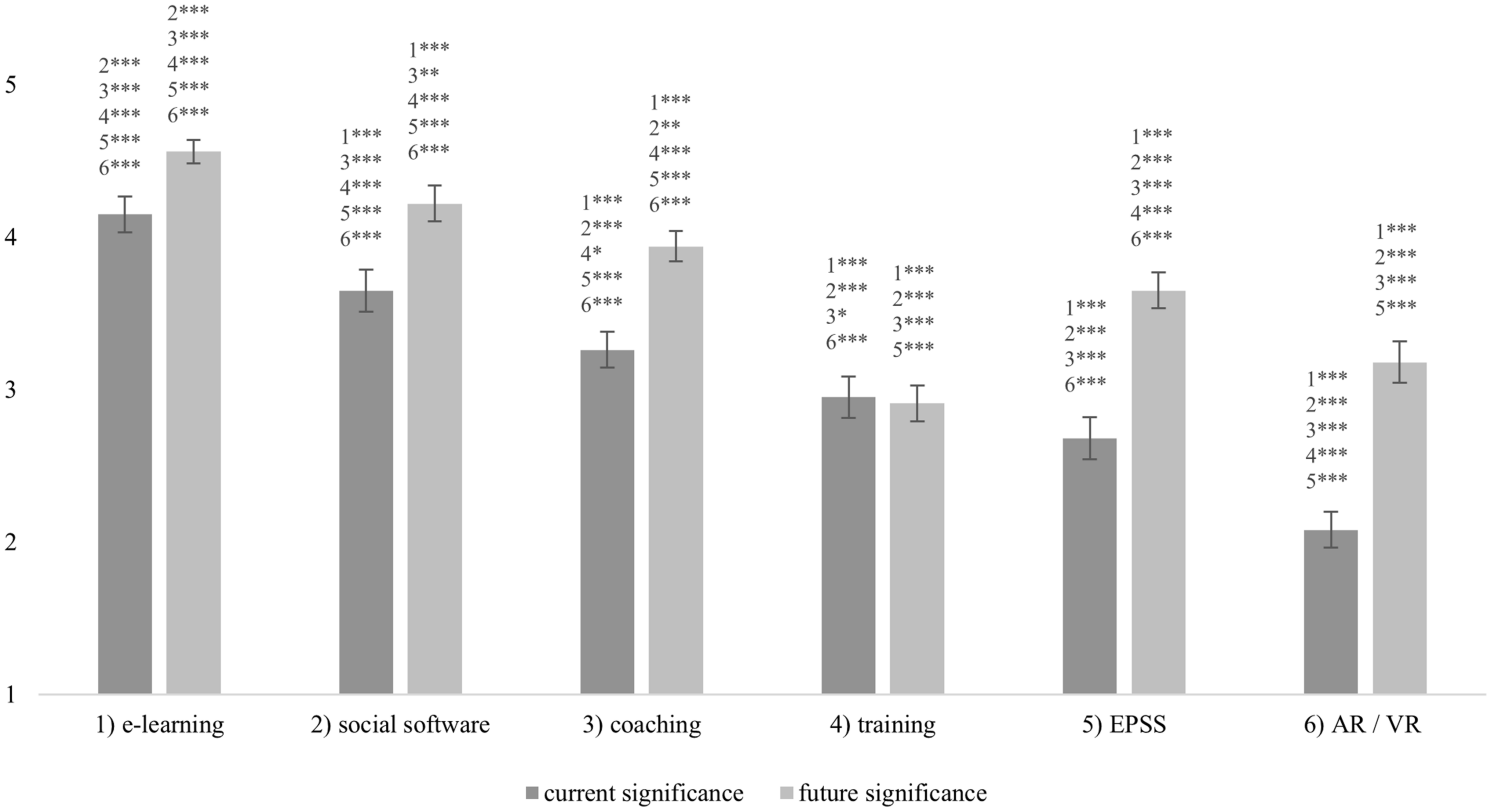
Current and future significance of different learning measures for employees as rated by HR employees. Significant differences, means, and confidence intervals. *N* = 299. Scale: 1 = irrelevant, 3 = partly relevant, 5 = very relevant. ^*^p < 0.05, ^**^p < 0.01, ^***^p < 0.001.

HR employees rated the current and future significance of six different learning measures for employees. A one-way repeated measures ANOVA with a Huynh–Feldt correction determined that mean current significance showed a statistically significant difference between the learning measures, *F*(4.151, 1236.97) = 150.821, *p* < 0.001, partial *η*^2^ = 0.34. Bonferroni-adjusted post-hoc analysis revealed several significant differences between the learning measures for current significance indicating substantial differences in perceived current significance between these learning measures. A second one-way repeated measures ANOVA with a Huynh–Feldt correction determined that mean future significance showed a statistically significant difference between the learning measures as well, *F*(4.087, 1217.91) = 139.604, *p* < 0.001, partial *η*^2^ = 0.32. Again, Bonferroni-adjusted post-hoc analysis revealed several significant differences between the learning measures for future significance. Again, this result shows that there are substantial differences in terms of future significance among these learning resources. Figure 3 shows all significant post-hoc results as well as the mean values and confidence intervals.

Results show the HR employees rated e-learning, social software, and coaching as the most significant measures. EPSS were currently considered less important which could be due to the limited scope of EPSS as compared to e-learning that can be applied for almost any learning goals. In the future, the same three learning measures are seen as most significant. But with a clearly greater increase in significance, EPSS will also play an important role in employee learning in the future.

### EPSS Advantages and Obstacles Concerning Their Implementation and Use (RQ2)

The participants selected from eight predefined potential advantages of EPSS those they considered to be applicable to their company. For potential obstacles concerning the implementation and use of EPSS, there were seven options to choose from. For both research questions, multiple answers were possible. Table 3 shows the proportions of participants that selected the given advantages.

**Table 3:** Perceived advantages of EPSS.

Increased employee efficiency due to reduced search and problem-solving time	Supplement to classroom trainings as an aid to the practical application of what has been learned	Reduction of search and problem-solving time	Reduction of helpdesk costs due to fewer queries about system operation	Facilitated communication of changes within software systems (e.g., cloud-based systems)	Supplement to classroom training for mixed learning scenarios	Support of employees during change processes	Substitute for classroom trainings
65	63	53	48	47	44	40	20

Percentage of participants that selected the respective advantage. 1,142 answers in total (multiple answers possible).

The most frequently selected advantages were (1) an increased employee efficiency, (2) the possibility to supplement face-to-face training, and (3) the reduction of search and problem-solving time. Thus, about two-thirds of the HR employees agreed that EPSS supports employee efficiency. Surprisingly, a learning-related advantage—the possibility to supplement face-to-face training by EPSS—takes second place before further performance-related advantages. Only 20% of the respondents considered EPSS a substitute for face-to-face training.

Table 4 shows the proportions of participants that selected the given obstacles concerning the implementation and use of EPSS. The results show that obstacles were seen in (1) a lack of resources to produce and maintain content, (2) too high technical effort, and (3) an already implemented, competing Learning Management Systems (LMS) as an alternative to an EPSS. Therefore, the HR employees considered monetary and technical efforts to be the biggest barriers to the implementation of EPSS, while acceptance problems by employees or work councils were expected by a small percentage of respondents. Altogether, the agreement with advantages (see Table 3) of EPSS significantly outweighed the agreement with disadvantages and obstacles.

**Table 4:** Perceived obstacles concerning the implementation and use of EPSS.

My company does not have the resources to produce a large amount of learning and support materials for our employees or keep it up to date.	The technical effort for such a system seems too high to me.	My company already has a Learning Management System. A second system to access learning content does not make sense to me.	The costs for the acquisition of EPSS offers or content from external providers seems too high to me.	The information provided will rarely match the actual questions.	A digital help system will find little or no acceptance among employees.	I think that our works council or our employee representatives would not accept such a system. (This may or may not apply to you, depending in which country you are working.)
34	33	33	32	25	17	14

Percentage of participants that selected the respective obstacle. 564 answers in total (multiple answers possible).

### Availability and Frequency of Use of Problem-Solving Activities (RQ3)

**Figure 4:**
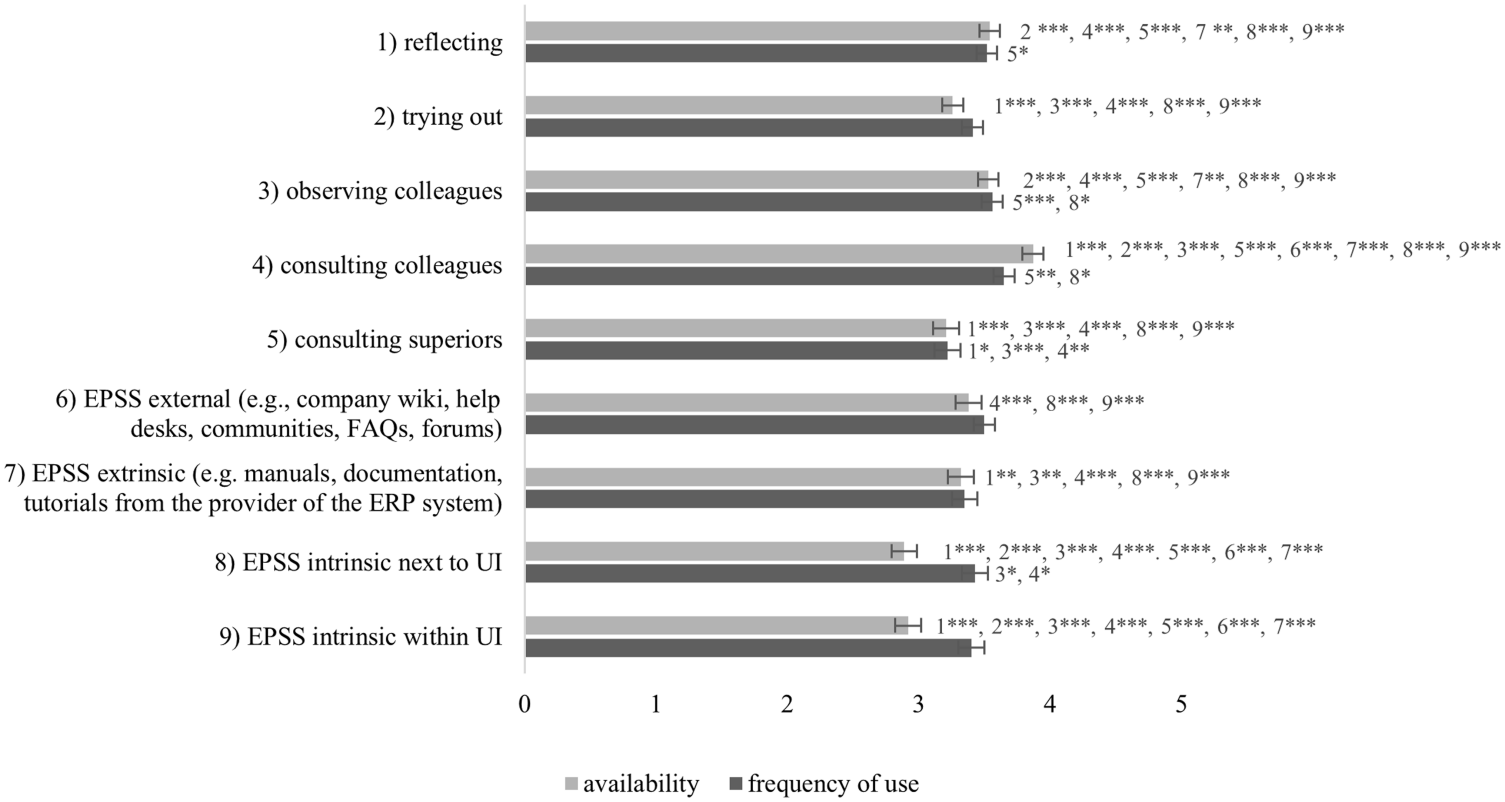
Significant differences, means, and confidence intervals of the availability and frequency of use of different problem-solving activities for ERP-related problems. Scale: 1 = not agree, 3 = partly, 5 = strongly agree. ^*^p < 0.05, ^**^p < 0.01, ^***^p < 0.001.

Based on study 2, Figure 4 shows to which degree different problem-solving activities are available to the surveyed ERP users and how frequently they use these activities. A one-way repeated measures ANOVA with a Greenhouse–Geisser correction determined that mean availability showed a statistically significant difference between the activities, *F*(5.92, 3709.56) = 66.74, *p* < 0.001, partial *η*^2^ = 0.10. Bonferroni-adjusted post-hoc analysis revealed several significant differences between the activities for availability. The differences between these groups can be interpreted as substantial. A second one-way repeated measures ANOVA with a Huynh–Feldt correction determined that mean frequency of use showed a statistically significant difference between the activities as well, *F*(7.04, 1245.80) = 5.42, *p* < 0.001, partial *η*^2^ = 0.03. Again, Bonferroni-adjusted post-hoc analysis revealed several significant differences between the activities for frequency of use, which are substantial differences. All significant post-hoc results are displayed in Figure 4.

Unsurprisingly, reflecting on one’s own as well as consulting and observing colleagues were perceived as the most available activity and were also used most frequently when confronted with ERP-related problems, however with less significant differences. External and extrinsic types of EPSS are already available to many users, while intrinsic EPSS are less often available. However, when available, they are used quite often, but only for extrinsic EPSS with information presented next to the user interface (UI) with few significant differences.

### Differences Between the ERP User Types in Terms of Availability and Frequency of EPSS Use (RQ4)

Two one-way MANOVAs were calculated to address RQ4. The first MANOVA was performed to determine the effect of ERP user types on the availability of EPSS. The test revealed statistically significant differences between the ERP user types on the combined dependent variables [*F*(12, 1,688) = 3.247, *p* < 0.001, Wilks’ Λ = 0.941, partial *η*^2^ = 0.020]. Follow-up univariate one-way ANOVAs were performed with Bonferroni adjustment due to alpha error inflation. Statistically significant differences were found for the availability of external EPSS and extrinsic EPSS with small effect sizes each. Tukey post-hoc tests showed that the group of administrators and SAP consultants has external EPSS more often available than end users, and extrinsic EPSS significantly more often available than occasional users and end users (Table 5). All other pairwise comparisons were not statistically significant.

**Table 5:** Descriptive statistics and MANOVA results among the four ERP user types for the availability of EPSS.

	*Occasional user (n = 181)*		*End user (n = 316)*		*Expert (n = 91)*		*Administrator or SAP consultant (n = 57)*		*F*	*p*	$\eta_{\rho }^{2}$	*Significant Tukey HSD*
	*M*	*SE*	*M*	*SE*	*M*	*SE*	*M*	*SE*				
External EPSS	3.48	0.09	3.24	0.07	3.40	0.13	3.91	0.16	5.29	0.001	0.024	4 > 2
Extrinsic EPSS	3.16	0.09	3.25	0.07	3.54	0.12	3.90	0.16	7.27	<0.001	0.033	4 > 1 4 > 2
Intrinsic EPSS information next to UI	2.79	0.09	2.81	0.07	3.21	0.13	3.11	0.17	3.33	0.019	0.015	
Intrinsic EPSS information within UI	2.85	0.09	2.84	0.07	3.13	0.13	3.28	0.17	3.17	0.024	0.015	

*N* = 645. 1 = occasional user; 2 = end user; 3 = expert; 4 = administrator or SAP consultant.

The second one-way MANOVA investigated the effect of ERP user types on the frequency of EPSS use. We only used a subset of 286 participants because the frequency of use was only asked for if the respective problem-solving activity was available. There are statistically significant differences between the ERP user types on the combined dependent variables [*F*(12, 738) = 2.055, *p* < 0.05, Wilks’ Λ = 0.917, partial *η*^2^ = 0.029] due to differences in the use of external EPSS with a small effect size. Follow-up univariate one-way ANOVAs with Bonferroni adjustment showed that the frequency of use of external EPSS differed statistically significantly between the user groups [*F*(3, 282) = 6.417, *p* < 0.001, partial *η*^2^ = 0.061]. Tukey post-hoc tests showed that administrators and SAP consultants (*M* = 4.18, *SE* = 0.16) use external EPSS significantly more often compared to occasional users (*M* = 3.37, *SE* = 0.10), *p* < 0.001, end users (*M* = 3.64, *SE* = 0.08), *p* < 0.05, and experts (*M* = 3.64, *SE* = 0.13), *p* < 0.05. All other pairwise comparisons were not statistically significant.

### Predictors of the Frequency of EPSS Use (RQ5)

A hierarchical multiple regression analysis was calculated in order to answer RQ5. Since not all respondents provided information on all investigated predictors, a subset of 568 participants was used. For each participant, the highest rating of frequency of EPSS use across all problem-solving activities including EPSS served as the dependent variable. Predictors were added in the course of five steps. In the first step, self-assessed ERP skills and the ERP user types were added as user characteristics. ERP user types were included by dummy coding (0/1) for each ERP user type with the group of administrators and SAP consultants as the reference group. In the second step, task characteristics regarding job demands and job control were added. Step three comprised the inclusion of the availability of the respective EPSS with the highest rating of frequency of use. This addresses the availability of the respective problem-solving activity. In step 4, we added the big five and proactive personality as personality traits. In the last step, team psychological safety as well as geographical separation were included. These are aspects regarding the social resources of a person and its working place. The results of the regression analysis are shown in Table 6. The correlation table for all variables included in the hierarchical regression can be found in the Supplementary Materials.

**Table 6:** Hierarchical multiple regression analysis summary for the MAX frequency of EPSS use.

	Model 1		Model 2		Model 3		Model 4		Model 5	
Variable	*β*	*B*	*β*	*B*	*β*	*B*	*β*	*B*	*β*	*B*
Self-assessed ERP skills	0.09	0.07	0.08	0.07	−0.05	−0.04	−0.04	−0.03	−0.05	−0.04
Occasional user	−0.73^***^	−0.33	−0.64^***^	−0.29	−0.52^***^	−0.23	−0.50^***^	−0.23	−0.51^***^	−0.23
End user	−0.68^***^	−0.34	−0.55^***^	−0.27	−0.39^**^	−0.19	−0.38^**^	−0.19	−0.38^**^	−0.19
Expert	−0.44^**^	−0.15	−0.41^*^	−0.14	−0.29^*^	−0.10	−0.31^*^	−0.11	−0.30^*^	−0.10
Task variety			0.11	0.08	0.14	0.10	0.14	0.10	0.14	0.10
Complexity			−0.21^***^	−0.20	−0.21^***^	−0.20	−0.19^***^	−0.18	−0.20^***^	−0.19
Problem-solving demands			0.15	0.12	0.18^**^	0.14	0.14	0.10	0.14	0.11
Information-processing requirements			−0.05	−0.04	−0.22^**^	−0.15	−0.20^*^	−0.13	−0.20^*^	−0.14
Autonomy			0.03	0.02	−0.06	−0.05	−0.08	−0.06	−0.10	−0.08
Availability for MAX frequency of EPSS use					0.59^***^	0.46	0.60^***^	0.47	0.59^***^	0.47
Neuroticism							0.01	0.01	0.02	0.02
Extraversion							0.06	0.06	0.06	0.06
Openness							0.08	0.07	0.08	0.07
Agreeableness							−0.10	−0.08	−0.12^*^	−0.10
Conscientiousness							−0.03	−0.03	−0.03	−0.02
Proactive personality							0.02	0.01	0.00	0.00
Team psychological safety									0.11	0.09
Geographical separation									0.08	0.04
*R* ^2^	0.062		0.125		0.301		0.313		0.318	
*F*	9.34^***^		8.89^***^		24.08^***^		15.74^***^		14.32^***^	
∆*R*^2^	0.062		0.063		0.176		0.012		0.006	
∆*F*	9.34^***^		8.05^***^		140.90^***^		1.59		2.33	

MAX frequency of EPSS use = highest frequency of use across all problem-solving activities including EPSS. Availability for MAX frequency of EPSS use = availability of the problem-solving activity with the highest frequency of use across all problem-solving activities including EPSS. *N* = 568.

* *p* < 0.05;

** *p* < 0.01;

*** *p* < 0.001.

The user characteristics contributed significantly to the regression model and explained 6.2% of the variance in the frequency of EPSS use. The inclusion of the job characteristics in step 2, *F*(5, 561) = 8.054, *p* < 0.001, as well as the inclusion of the availability of the respective EPSS in step 3, *F*(1, 560) = 140.901, *p* < 0.001, lead to significant increases in the explained variance of 6.3% respective 17.6%. Adding the personality traits in step 4, *F*(6, 554) = 1.587, *p* = n.s., and the aspects regarding the social resources in step 5, *F*(2, 552) = 2.332, *p* = n.s., did not improve the explained variance in the frequency of EPSS significantly. Of these variables only agreeableness (*β* = −0.12, *p* < 0.05) was a significant predictor of frequency of EPSS use. Both models were still statistically significant, *R*^2^ = 0.313, *F*(16, 554) = 15.740, *p* < 0.001, adjusted *R*^2^ = 0.293, respective *R*^2^ = 0.318, *F*(18, 552) = 14.318, *p* < 0.001, adjusted *R*^2^ = 0.296. However, as there were no significant increases in the explained variance, the variables included in the last two steps have only a very small influence on the frequency of EPSS use. Referring to the significant predictors, EPSS availability was a positive and also the strongest predictor of EPSS use. Furthermore, the dummy variables for the ERP user types were significant predictors and indicate that more intensive ERP users also use EPSS more frequently, while the self-assessed ERP skills were not significant. In addition, agreeableness as well as the task complexity and information-processing requirements showed small negative effects.

### Perceived Usefulness of EPSS Characteristics (RQ6)

**Figure 5:**
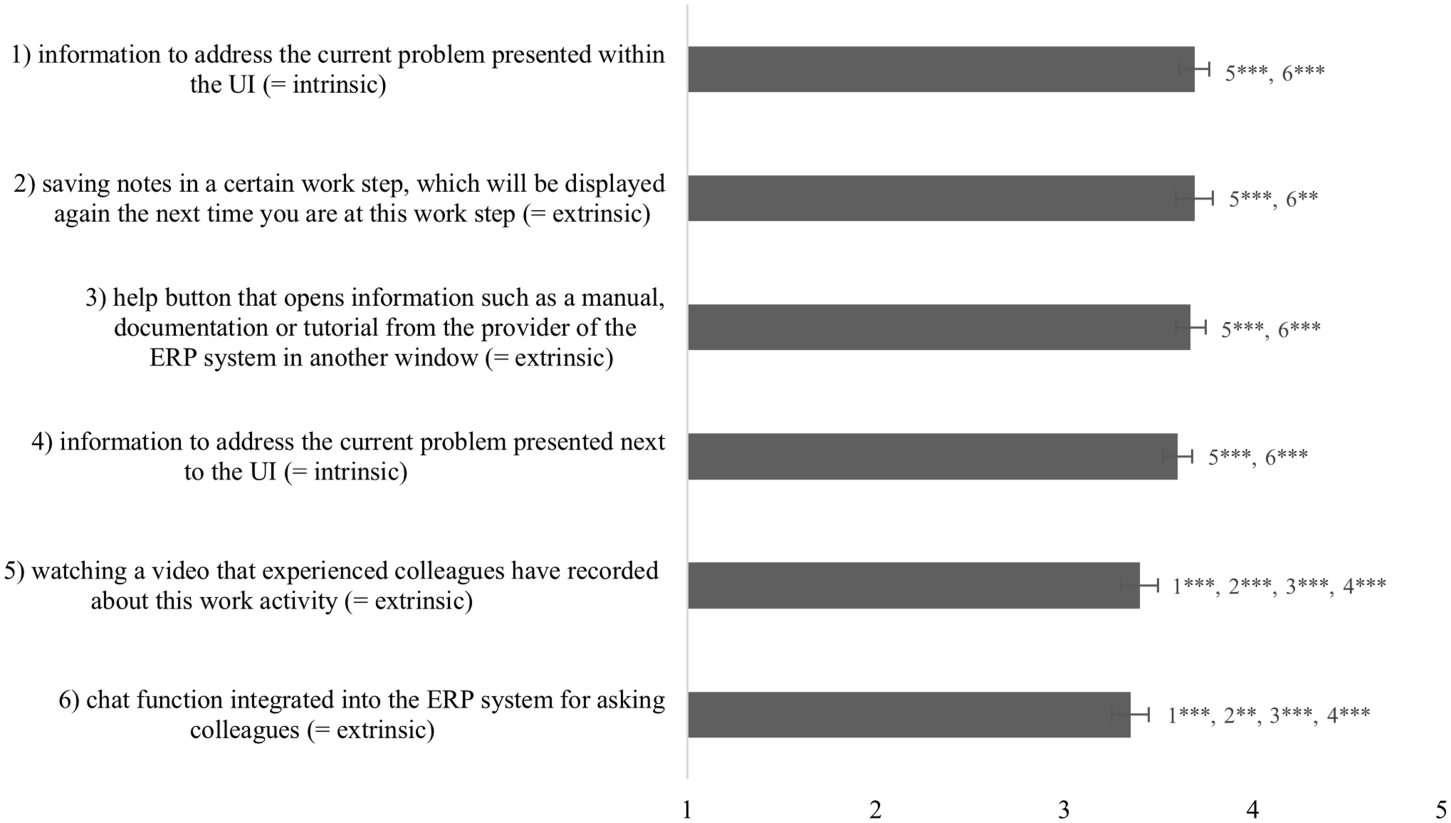
Significant differences, means and confidence intervals of the perceived usefulness of EPSS characteristics. Scale: 1 = not helpful at all, 3 = partly helpful, 5 = very helpful. ^*^p < 0.05; ^**^p < 0.01; and ^***^p < 0.001.

The ERP users indicated the perceived usefulness of different EPSS characteristics for solving ERP-related problems (Figure 5). A one-way repeated measures ANOVA with a Huynh–Feldt correction determined that mean usefulness showed a statistically significant difference between the EPSS characteristics, *F*(3.86, 2488.19) = 21.18, *p* < 0.001, partial *η*^2^ = 0.03. Bonferroni-adjusted post-hoc analysis revealed several significant differences between the EPSS characteristics for usefulness. These are substantial differences that can be interpreted. Significant differences are also displayed in Figure 5.

All EPSS characteristics presented to the ERP users were rated as useful but only on a medium level. The displaying of context-sensitive information within the UI, the possibility to save one’s own notes but also displaying information in an extra window were considered to be slightly more useful. As theoretically already expected, there was no general preference for intrinsic over extrinsic characteristics.

In order to investigate if the ERP user types differ in their assessment of the perceived usefulness, a one-way MANOVA was performed. The analysis revealed statistically significant differences between the ERP user types on the combined dependent variables [*F*(18, 1,802) = 1.776, *p* < 0.05, Wilks’ Λ = 0.951, partial *η*^2^ = 0.016] but no significant results for the follow-up univariate one-way ANOVAs with Bonferroni adjustment were found. This indicates that there are no substantial differences between the user groups that can be reported.

## Discussion

Electronic Performance Support Systems (EPSS) are considered to support problem-solving and learning in the context of complex software tools, such as Enterprise Resource Planning (ERP) systems. In two survey studies, we asked 301 HR employees about their perception of EPSS as a learning measure in companies and 652 ERP users about their perception of EPSS when solving ERP-related problems. In general, EPSS can be viewed from two perspectives. On the one hand, EPSS can be viewed as a technological resource created to support employees’ performance, problem-solving, and learning. This is a more general view on EPSS that includes, for example, how they are designed and supplied. On the other hand, EPSS can be considered regarding their actual use for problem-solving and potentially informal learning. Study 1 addressed the former perspective, while study 2 was based mostly on the latter perspective.

### EPSS as a Trend in In-Company Learning Support

Asked about trends in in-company learning measures (RQ1), the HR employees rated e-learning, social software. and coaching as the most significant measures. EPSS were currently considered less important which could be due to the limited scope of EPSS as compared to e-learning that can be applied for almost any learning goals. Another reason might be the quite low penetration rate of EPSS in companies, which is also evident in the survey of ERP users in study 2. Furthermore, EPSS are primarily designed to support performance and only as a by-product do they also support learning. Thus, they are a less obvious learning measure compared to e-learning. Still, HR employees assign high future significance to EPSS.

Asked about advantages and obstacles concerning the implementation and use of EPSS (RQ2), the HR employees selected significantly more pros than cons which again confirms their positive attitude toward EPSS. The most frequently selected advantages were (1) an increased employee efficiency, (2) the possibility to supplement face-to-face training, and (3) the reduction of search and problem-solving time. Obstacles were seen in (1) a lack of resources to produce and maintain content, (2) too high technical effort, and (3) an already implemented, competing Learning Management Systems (LMS) as an alternative to an EPSS. Anticipated acceptance problems on part of the employees or work councils played a minor role.

### EPSS Use as an Activity for Solving ERP-Related Problems

Everyday problem-solving and informal learning go hand in hand. Starting from a classification of problem-solving approaches in the workplace (see Table 1), we developed a Model of Informal Workplace Learning Through Problem-Solving (see Figure 2), which integrates assumptions of Tynjälä’s (2013) 3-P model, the JDCS model (Karasek, 1979; Johnson and Hall, 1988; Karasek and Theorell, 1990), the Approaches to Problem-Solving in the Workplace (Rausch, 2011; Rausch et al., 2015), the Technology Acceptance Model (Davis et al., 1989; Venkatesh and Bala, 2008), and the Affective Events Theory (AET) by Weiss and Cropanzano (1996). When confronted with an ERP-related problem, available personal, social, and technological resources are assessed, more or less consciously, regarding their potential contribution to the solution (i.e., usefulness) and regarding the effort required (i.e., ease of use). Ideally, EPSS provide useful and easy-to-use support that fosters problem-solving and learning. Therefore, EPSS conserve (social) resources in the short term (i.e., experts’ working time, time spent on the problem) and expand personal resources in the long term (i.e., competence development). However, empirical research on EPSS use is scarce. While study 1 covered the potential that EPSS could have for competence development and workplace learning, study 2 investigated the contextual factors and individual factors/personal resources, including possible problem-solving activities (e.g., EPSS use), as well as the components of the interpretation and activities’ frequency of use.

Regarding the availability and frequency of use of problem-solving activities (RQ3), the ERP users reported that consulting colleagues is the most frequently available and most frequently used activity, which was also reported in a diary study by Rausch et al. (2015). Reflecting is the second most frequently used activity, although it is assumed that reflecting on a problem is included in any problem-solving process, at least to some degree. However, high time pressure or low self-efficacy could lead to shorter reflection. Observing colleagues was also rated as a frequently available and well-used activity. When colleagues show a problem solution, it can be assumed that they were asked beforehand. External and extrinsic types of EPSS are also available and used similarly frequently while intrinsic EPSS are less frequently available but if so, they are used intensively. This shows that EPSS, regardless of their categorization, are generally perceived as useful and easy to use. In line with our expectation, the results do not indicate a fundamental superiority of one EPSS type over another.

Investigating differences between the user types (RQ4) revealed that the group with the supposedly highest skills, administrators or SAP consultants, have external (i.e., company wiki, help desks, communities, FAQs, and forums) and extrinsic EPSS (i.e., manuals, documentations, and tutorials from the provider of the ERP system) more often available than other user groups and they also use external EPSS more often than other user groups. This could be related to the fact that forums and question-and-answer websites, for instance, fall into the category of external EPSS and that these are suitable for very specific and complex problems and questions, especially from experienced ERP users. It is conceivable that experts, in particular, may even only find help for their complex problems in such external EPSS because there is not enough expertise in their own team. In software programming, for instance, a lot of experts use Stack Overflow (a question-and-answer website for professional programmers) for their more complex problems.

Addressing contextual and individual/personal antecedents of the frequency of EPSS use (RQ5), a hierarchical multiple regression revealed that personality as well as aspects regarding the social resources were only less relevant for predicting frequency of EPSS use. EPSS availability was the strongest predictor, which is, of course, not surprising. Regarding further contextual factors, complexity and information-processing requirements were significant negative predictors of EPSS use. This would be in line with the results presented above that indicated a high frequency of use of external EPSS by experts with probably more complex problems. Regarding user characteristics, the ERP user role explained additional variance. This result also confirms the above findings that the most experienced user group uses EPSS rather frequently, due to the availability of external EPSS also in the case of more complex problems. The self-assessed ERP skills were not a predictor of EPSS use. Regarding the general personality traits, only agreeableness was a negative predictor which is not in line with the results by Devaraj et al. (2008) who found agreeableness to be a positive predictor of technology acceptance. Since people high in agreeableness tend to cooperate (McCrae and Costa, 1987), they may also tend to consult others instead of using the EPSS. However, the same could be expected for extraverted people but was not found in our data. Altogether, general personality traits do not seem to play an important role in the use of EPSS. The same is true for team psychological safety and a person’s geographical separation from the team as potential social resources.

Asked for the most favored characteristics of EPSS (RQ6), ERP users particularly valued context-sensitive information displayed within the UI of the ERP software, the possibility to save one’s own notes within the system, and information displayed in an extra window. However, all EPSS characteristics were assessed as only moderately useful with small mean differences and participants did not receive detailed explanations or demos to illustrate the different characteristics. Therefore, the results should be interpreted with caution and further empirical results from the actual use of these characteristics are necessary. The possibility to watch a video that experienced colleagues have recorded about this work activity was rated as partly useful but only in fifth place. This is surprising as several authors emphasize the importance of employees’ possibility to document and share their knowledge for colleagues (Gorecky et al., 2014; Ley et al., 2014). Perhaps the item was not worded precisely enough. Furthermore, results showed that there were no significant differences found between the ERP user groups’ assessment of the usefulness of the different EPSS characteristics.

Altogether, HR employees attach a greater significance to EPSS in the future. They see an increased efficiency and a supplement to face-to-face training as the biggest advantages. External EPSS, including Web 2.0 services and applications, and extrinsic EPSS types are already available quite often, while intrinsic EPSS are less common. However, all EPSS types are actively used when available. The ERP users indicated context-sensitive information, integrated into the ERP system’s UI, the option to save one’s own notes for similar cases in the future, and information displayed in an extra window as more useful EPSS characteristics. In general, EPSS are more often available for more experienced users, such as ERP administrators and SAP consultants; and this user group uses external EPSS, such as company wikis, help desks, communities, FAQs, and forums more often than others. Still, consulting and observing colleagues are more common approaches when being confronted with ERP-related problems.

Regarding the developed Model of Informal Workplace Learning Through Problem-Solving, the results of study 2 found some of the individual factors/personal resources and contextual factors to be significantly related to EPSS use for solving ERP-related problems. Furthermore, the various activities for problem-solving generally available in the workplace according to the model could also be identified as empirically relevant. In addition, study 1 confirms the potential of EPSS for employee workplace learning, that is proposed by the model.

### Limitations and Future Research

First of all, as the participants of both survey studies participated voluntarily, the results could be biased due to self-selection (Bickman and Rog, 2009; Henry, 2009). Furthermore, the participants of both survey studies were mainly from Germany, which also limits the generalizability of the results (Bickman and Rog, 2009). Moreover, given the cross-sectional study design, causal interpretations should be treated with caution (Bickman and Rog, 2009; Kelley and Maxwell, 2019).

Regarding study 2, we included a measure for the big five personality traits based on Saucier’s (1994) Mini Markers and their German version by Weller and Matiaske (2009). However, we did not use separate items for each adjective, but to reduce participant burden, we used an array of adjectives in one item for each personality trait. This may have resulted in less accurate measurement of the big five personality traits, which could have affected the regression results by either overestimating or underestimating the effects. Furthermore, for measuring the availability and frequency of use of EPSS, as well as the perceived usefulness of EPSS characteristics, we generally referred to ERP-related problems in the workplace without specifying them in more detail. This allowed each participant to imagine a different ERP-related problem. It might be possible that depending on the problem imagined, the items on availability, frequency of use, and usefulness were rated differently. This may have negatively affected the precision and reliability of the results and further limited the generalizability of the results. In addition, as already mentioned, the EPSS characteristics and their function were only described verbally without seeing them in a system. This was very hypothetical and gave participants room for interpretation. This, again, may have led to less precise assessments of usefulness, on the one hand, and may limit the generalizability of the results on the other. Another limitation of our research is that we did not include the possibility that EPSS can proactively indicate a problem to the user, and only then does the user become aware of the problem. Such a feature would be feasible with AI. A further limitation of the study is that we did not investigate all components of the developed model. The components of contextual factors, individual factors/personal resources, interpretation, and problem-solving activities are covered, however not the actual outcomes as well as users’ emotional experiences.

Addressing the above limitation, future research should also investigate actual EPSS use near the process, for instance, by using research diaries. They measure not only closer to the object under investigation but also reduce memory bias of retrospective questionnaires (Bolger et al., 2003; Ohly et al., 2010; Rausch et al., 2017). Furthermore, future studies could also investigate proactive EPSS as mentioned above. Regarding the developed model, further studies addressing the assumed impact of the individual factors/personal resources and contextual factors should be conducted, as only some aspects of these factors were found to be empirically related to EPSS use so far. Moreover, the link between EPSS use, respective the use of information sources in general, and learning as well as the influence of emotional experiences were not investigated empirically yet. Thus, these variables should also be included in future empirical studies.

### Practical Implications

Our findings suggest a positive impact of EPSS on employee performance in solving ERP-related problems, and also indicate that EPSS might positively influence employees’ informal learning on some aspects. These results can be relevant for ERP system vendors as well as companies using ERP systems. For both, it can be recommended to integrate different EPSS characteristics into ERP systems. For vendors, this primarily includes content on standard processes and applications, as well as general content that supports rapid onboarding of new employees into the system. For the vendors, this can also serve as an USP. Companies that use ERP systems can then augment this content, for example, with more detailed help on specific processes or error-prone items as well as special aspects and areas of application. Although the possibility to watch videos that were recorded by experienced colleagues was not rated as especially helpful in our study, in our opinion, this is nevertheless a possibility that companies should take a closer look at. Our results suggest that external EPSS can be especially important for more experienced users. Here we assume that social communities, implemented through social technology, are of central importance. These can be established and explicitly promoted within the company. Furthermore, an additional link to user and competence profiles is conceivable. This would allow for the incorporation of prior knowledge and training already completed to provide context-specific and tailored support.

In line with Clark (1992), we assume that EPSS only foster particular skills, namely, the use of software tools, which are only one part of a broader set of professional competences that are required today (Rausch and Wuttke, 2016). Therefore, interaction with experienced coworkers and participation in collaborative problem-solving will still play an important role in workplace learning and socialization (Gery, 1991; Billett, 2001). It is not a question of either EPSS or other learning resources, but of an appropriate combination of different opportunities to learn in the workplace.

## Data Availability Statement

The raw data supporting the conclusions of this article will be made available by the authors, without undue reservation.

## Author Contributions

All listed authors have made a substantial, a direct, and an intellectual contribution to this work and approved it for publication.

## Funding

This research was partly funded by SAP SE. The funder was not involved in the study design, collection, analysis, interpretation of data, the writing of this article or the decision to submit it for publication. Open access publication fee was partly funded by the Open Access Publishing Fund at the University of Mannheim, Germany.

## Conflict of Interest

The authors declare that the research was conducted in the absence of any commercial or financial relationships that could be construed as a potential conflict of interest.

## Publisher’s Note

All claims expressed in this article are solely those of the authors and do not necessarily represent those of their affiliated organizations, or those of the publisher, the editors and the reviewers. Any product that may be evaluated in this article, or claim that may be made by its manufacturer, is not guaranteed or endorsed by the publisher.
