# Supplementary material for: Problem-Solving and Tool Use in Office Work: The Potential of Electronic Performance Support Systems to Promote Employee Performance and Learning
Source: Front Psychol. 2022 Apr 29;13:869428. doi: 10.3389/fpsyg.2022.869428 (PMC9102809; doi:10.3389/fpsyg.2022.869428)
Supplement: Supplementary file 1 [file Data_Sheet_1.docx]

Supplementary Material

**Survey items used in study 1**

*Significance of different learning measures for employees*

What is the significance of the following measures for employee learning in your company at present? (from 1 = *irrelevant* to 5 = *very relevant*)

- Classroom training (seminars and training courses lasting several hours to several days)
- Coaching (targeted support and advice from other people)
- E-learning (Web-based trainings, MOOCs, Webinars, virtual classrooms)
- Augmented Reality / Virtual Reality
- Social software (communication channels between employees, chats, forums, yellow pages etc.)
- Electronic Performance Support (context-specific help for user software, e.g., in text editing programs or in the ERP system)

What is the significance of the following measures for employee learning in your company in the future (in the next 3 to 5 years) (from 1 = *irrelevant* to 5 = *very relevant*)?

- Classroom training (seminars and training courses lasting several hours to several days)
- Coaching (targeted support and advice from other people)
- E-learning (Web-based trainings, MOOCs, Webinars, virtual classrooms)
- Augmented Reality / Virtual Reality
- Social software (communication channels between employees, chats, forums, yellow pages etc.)
- Electronic Performance Support (context-specific help for user software, e.g., in text editing programs or in the ERP system)

*Advantages and obstacles concerning the implementation and use of EPSS*

What advantages do you see in the launch or use of Electronic Performance Support Systems (EPSS) in your company?

- Substitution for classroom trainings
- Supplement to classroom trainings as an aid to the practical application of what has been learned
- Supplement to classroom training for mixed learning scenarios
- Reduction of helpdesk costs due to fewer queries about system operation
- Reduction of search and problem solving time
- Increased employee efficiency due to reduced search and problem solving time
- Support of employees during change processes
- Facilitated communication of changes within software systems (e.g., cloud-based systems)

What obstacles do you see for the launch or use of Electronic Performance Support Systems (EPSS) in your company?

- A digital help system will find little or no acceptance among employees.
- The information provided will rarely match the actual questions.
- The technical effort for such a system seems too high to me.
- I think that our works council or our employee representatives would not accept such a system. (This may or may not apply to you, depending in which country you are working.)
- My company does not have the resources to produce a large amount of learning and support materials for our employees or keep it up to date.
- The costs for the acquisition of EPSS offers or content from external providers seems too high to me.
- My company already has a Learning Management System. A second system to access learning content does not make sense to me.

**Survey items used in study 2**

*ERP user type*

What kind of ERP user would you most likely describe yourself as?

- Occasional user (I use the ERP system, for example, to have my vacation approved, to submit a travel request, or for actions that only occur quarterly or once a year.)
- End user (I regularly use the ERP system as part of my normal work activities.)
- Expert (I own the Key User role and/or I am the person in my team or department who is contacted for questions regarding the ERP system.)
- Administrator or SAP consultant (As part of my job, I am responsible for the configuration and adaptation of the ERP system. Or: Within the scope of my work, I advise other companies regarding SAP software.)

*Self-assessed skills using the ERP system*

Please assess to what extent the following statements apply to you and your use of the ERP system (from 1 = *not agree at all* to 5 = *strongly agree*). When using the ERP system…

- …I feel very safe with the applications I need regularly.
- …I complete my tasks very quickly.
- …I know my way around very well.

*Proactive personality*

Please assess to what extent the following statements apply to your behaviour at work (from 1 = *not agree at all* to 5 = *strongly agree*).

- When I see something I think is bad, I try to change it.
- I like to fight for my ideas, even against the resistance of others.
- I am always looking for ways to make things better.
- If I have a problem, I take care of it immediately.

*Big five personality traits*

Please assess how well the following adjectives describe your personality (from 1 = *not agree at all* to 5 = *strongly agree*).

- easily provoked, sensitive, touchy, moody
- extroverted, talkative, communicative, cheerful
- innovative, creative, educated, well-read
- helpful, kind, sympathetic, warm-hearted
- careful, tidy, conscientious, systematic

*Characteristics of the work task*

Please now assess to what extent the following statements apply to your current job and workplace (from 1 = *not agree at all* to 5 = *strongly agree*). At my workplace…

- …I do a lot of different things.
- …my job requires that I only do one task or activity at a time.
- …my job requires me to monitor a great deal of information.
- …my job involves solving problems that have no obvious correct answer.
- …I can plan how I do my work.
- …I do something new every now and then.
- …the tasks of my job are simple and uncomplicated.
- …my job requires that I engage in a large amount of thinking.
- …my job requires me to be creative.
- …I can make a lot of decisions on my own.
- …I have to deal with a variety of tasks.
- …almost anyone could do my work without much training.
- …my job requires me to keep track of more than one thing at a time.
- …my job often involves dealing with problems that I have not met before.
- …I have significant autonomy in making decisions.
- …my work is very varied.
- …my work is not very demanding.
- …my job requires me to process a lot of information.
- …my job requires unique ideas or solutions to problems.
- …I can make decisions about what methods I use to complete my work.

*Geographical separation*

Are you usually geographically separated from the core of your team (e.g., other site or home office)? If you have been in the home office for all or most of the time due to the Corona pandemic, please click "yes" (yes/no).

*Team psychological safety*

Please assess to what extent the following statements apply to your team (from 1 = *not agree at all* to 5 = *strongly agree*). In my team…

- …it is easy to speak up about what is on your mind.
- …people are usually comfortable talking about problems and disagreements.
- …people are eager to share information about what does and doesn’t work.
- …it is often held against you if you make a mistake.

*Availability of problem-solving activities* and *frequency of use of problem-solving activities*

Imagine the following situation: You have a problem in the ERP system. Please answer the following questions (from 1 = *not agree at all* to 5 = *strongly agree*). If the possibility is at least partly available, you are then asked how often you use the possibility (“I often use this possibility.”; from 1 = *not agree at all* to 5 = *strongly agree*). At my workplace, if I have problems with the ERP system, I basically have the possibility to…

- …think longer in order to come to a solution by myself.
- …keep trying until I find a solution myself.
- …watch colleagues who are solving such problems.
- …ask my colleagues for help.
- …ask my superior for help.
- …access internal company information sources (e.g., company wiki, help desks, communities, FAQs, forums).
- …access help integrated in the ERP system (e.g., manuals, documentation, tutorials from the provider of the ERP system).
- …use integrated help systems, which are displayed next to the user interface of the ERP system and support me specifically with my current problem.
- …use integrated help systems, which are displayed within the user interface of the ERP system and support me specifically with my current problem.

*Perceived usefulness of EPSS characteristics*

Please assess how helpful you find the following help offerings within an ERP system for solving a specific problem, regardless of whether these options are actually available to you at your workplace (from 1 = *not helpful at all* to 5 = *very helpful*). In the ERP system, you can…

- …click on a help button, which opens another window with information such as a manual, a documentation or a tutorial from the provider of the ERP system.
- ...use information provided next to the user interface of the ERP system to complete the current problem.
- …use information provided within the user interface of the ERP system to complete the current problem.
- …save your own notes in specific steps within the ERP system, which are displayed again when you reach this step the next time.
- …use an integrated chat function to ask colleagues.
- …watch videos that experienced colleagues in your company have recorded on their actions.

**Correlation table of hierarchical multiple regression variables (RQ5)**

|  | M | SD | 1 | 2 | 3 | 4 | 5 | 6 | 7 | 8 | 9 | 10 | 11 | 12 | 13 | 14 | 15 | 16 | 17 | 18 |
| --- | --- | --- | --- | --- | --- | --- | --- | --- | --- | --- | --- | --- | --- | --- | --- | --- | --- | --- | --- | --- |
| 1. MAX frequency of EPSS use | 3.73 | 1.00 |  |  |  |  |  |  |  |  |  |  |  |  |  |  |  |  |  |  |
| 2. Self-assessed ERP skills | 3.90 | 0.79 | 0.13** |  |  |  |  |  |  |  |  |  |  |  |  |  |  |  |  |  |
| 3. Occasional user | 0.28 | 0.45 | -0.11** | -0.25*** |  |  |  |  |  |  |  |  |  |  |  |  |  |  |  |  |
| 4. End user | 0.49 | 0.50 | -0.09* | -0.03 | -0.60*** |  |  |  |  |  |  |  |  |  |  |  |  |  |  |  |
| 5. Expert | 0.14 | 0.34 | 0.08* | 0.22*** | -0.25*** | -0.39*** |  |  |  |  |  |  |  |  |  |  |  |  |  |  |
| 6. Task variety | 3.94 | 0.70 | 0.14*** | 0.26*** | -0.05 | -0.08* | 0.03 |  |  |  |  |  |  |  |  |  |  |  |  |  |
| 7. Complexity | 3.60 | 0.96 | -0.19*** | 0.07 | -0.02 | 0.06 | -0.08* | 0.26*** |  |  |  |  |  |  |  |  |  |  |  |  |
| 8. Problem-solving demands | 3.63 | 0.77 | 0.20*** | 0.13** | 0.00 | -0.15*** | 0.04 | 0.69*** | 0.05 |  |  |  |  |  |  |  |  |  |  |  |
| 9. Information-processing requirements | 4.03 | 0.68 | 0.07* | 0.26*** | -0.02 | -0.07* | 0.02 | 0.71*** | 0.32*** | 0.59*** |  |  |  |  |  |  |  |  |  |  |
| 10. Autonomy | 3.66 | 0.76 | 0.19*** | 0.25*** | -0.03 | -0.13** | 0.08* | 0.59*** | -0.09* | 0.59*** | 0.42*** |  |  |  |  |  |  |  |  |  |
| 11. Availability for MAX frequency of EPSS use | 4.00 | 0.79 | 0.46*** | 0.33*** | -0.06 | -0.10* | 0.08* | 0.25*** | 0.06 | 0.20*** | 0.31*** | 0.26*** |  |  |  |  |  |  |  |  |
| 12. Neuroticism | 2.16 | 1.07 | 0.06 | -0.14*** | 0.03 | -0.09* | 0.06 | -0.12** | -0.35*** | -0.01 | -0.14** | -0.03 | -0.10* |  |  |  |  |  |  |  |
| 13. Extraversion | 3.39 | 1.01 | 0.15*** | 0.08* | 0.03 | -0.08* | 0.03 | 0.18*** | -0.14** | 0.18*** | 0.12** | 0.23*** | 0.11** | 0.13** |  |  |  |  |  |  |
| 14. Openness | 3.78 | 0.82 | 0.18*** | 0.19*** | 0.01 | -0.12** | 0.03 | 0.40*** | -0.05 | 0.41*** | 0.38*** | 0.39*** | 0.21*** | -0.13** | 0.22*** |  |  |  |  |  |
| 15.Agreeableness | 4.16 | 0.79 | 0.05 | 0.23*** | 0.06 | -0.08* | -0.04 | 0.29*** | 0.07* | 0.13** | 0.33*** | 0.22*** | 0.28*** | -0.22*** | 0.16*** | 0.29*** |  |  |  |  |
| 16. Conscien-tiousness | 4.05 | 0.90 | 0.01 | 0.24*** | -0.02 | 0.03 | -0.03 | 0.22*** | 0.08* | 0.07* | 0.29*** | 0.09* | 0.18*** | -0.16*** | 0.01 | 0.26*** | 0.35*** |  |  |  |
| 17. Proactive personality | 3.86 | 0.64 | 0.17*** | 0.35*** | -0.08* | -0.06 | 0.09* | 0.46*** | 0.03 | 0.44*** | 0.47*** | 0.45*** | 0.32*** | -0.12** | 0.22*** | 0.47*** | 0.35*** | 0.37*** |  |  |
| 18. Team psychological safety | 3.86 | 0.76 | 0.11** | 0.29*** | 0.03 | -0.06 | -0.02 | 0.37*** | 0.24*** | 0.23*** | 0.36*** | 0.34*** | 0.25*** | -0.34*** | 0.04 | 0.26*** | 0.35*** | 0.20*** | 0.38*** |  |
| 19. Geographical separation | 0.61 | 0.49 | 0.08* | -0.03 | 0.01 | -0.05 | 0.02 | -0.06 | -0.11** | 0.08* | -0.06 | 0.08* | 0.02 | 0.09* | 0.02 | 0.01 | 0.00 | -0.11** | -0.08* | -0.10** |

MAX frequency of EPSS use = highest frequency of use across all problem-solving activities including EPSS. Availability for MAX frequency of EPSS use = availability of the problem-solving activitiy with the highest frequency of use across all problem-solving activities including EPSS. N = 568. * p < 0.05, ** p < 0.01, *** p < 0.001
